# Giant Lipoma of the Arm Masquerading as an Accessory Breast: A Report of a Rare Case

**DOI:** 10.7759/cureus.45300

**Published:** 2023-09-15

**Authors:** Amit Kumar Yadav, Aditya Sharma, Ram Niwas Meena

**Affiliations:** 1 Department of General Surgery, Maa Vindhyavasini Autonomous State Medical College, Mirzapur, IND; 2 Department of General Surgery, Institute of Medical Sciences, Banaras Hindu University, Varanasi, IND

**Keywords:** tertiary healthcare center, lipomatous lesion, ectopic breast tissue, fine needle aspiration cytology (fnac), giant lipoma

## Abstract

Accessory breast is an extremely rare condition that develops in 0.4%-6% of females. It is primarily found in the axilla and is frequently misdiagnosed. It is usually bilateral and manifests during pregnancy or lactation as an asymptomatic tumor. The diagnosis of ectopic breast tissue is crucial because it is capable of undergoing the same pathological changes as normal breast tissue, including mastitis, fibrocystic changes of the breast tissue, and, in extreme cases, even malignancy.

We present the case of a 40-year-old female who presented with swelling in the left upper arm for the past eight years, which was associated with pain. Initially, accessory breast was kept as the clinical diagnosis. However, further imaging and histological analysis revealed it to be a giant lipoma of the upper arm.

## Introduction

A combination of more than two breasts in a human, with or without a nipple and areola, is known as having accessory breasts [1]. The basic embryonic milk lines, which run from the axilla to the groin, may be affected anywhere along their length [2]. The same physiological and pathological processes that affect the typically located breast, such as lactational transformation, fibroadenoma, and malignancies, may potentially occur in accessory breast tissue [3].

The occurrence of accessory breast tissue is uncommon. Additionally, if the swelling develops in the axilla or groin, it could be difficult to diagnose because it could clinically resemble a lymphoma or another type of lymphadenopathy [4]. We chose to report this case because of its rarity and propensity to present a clinical diagnostic challenge.

## Case presentation

A 40-year-old Asian female presented to the general surgery outpatient department with chief complaints of swelling over her left upper arm for eight years. The swelling was initially smaller in size and has gradually progressed to its present size. There was no previous history of a cough, fever, weight loss, night sweats, or any other constitutional symptoms except for mild dyspepsia.

She occasionally took unlabeled over-the-counter antacids for her dyspepsia as the symptoms manifested. She has never undergone surgery of any kind before. There was no addiction history. She reported having normal menstrual cycles and no major medical problems.

Except for the mild pallor, the rest of the examination was within normal limits. There was no generalized lymphadenopathy. On systemic examination, all the higher mental functions were intact; on auscultation, for the heart sounds, S1 and S2 were heard without any murmur, and bilateral air entry was present without any obvious added sounds. The abdomen examination was within normal limits. The genitourinary examination was also within normal limits.

**Figure 1:**
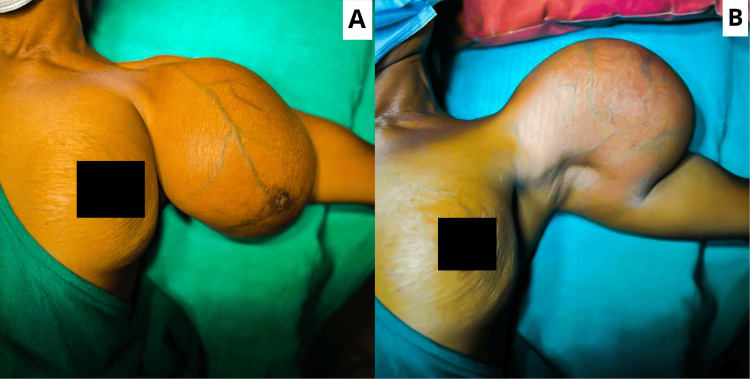
(A) A swelling of 20×15 cm is present over the left upper arm with venous engorgement and a scab mimicking the nipple-areola complex over the swelling. (B) The skin underlying the swelling was within the normal limits, and the axilla was also within normal limits.

On examination of the affected region, a swelling of 20×15 cm was present over the left upper arm without any overlying skin changes. Venous engorgement was present over the swelling, along with a scab in the middle mimicking the nipple-areola complex as shown in Figure 1.

**Figure 2:**
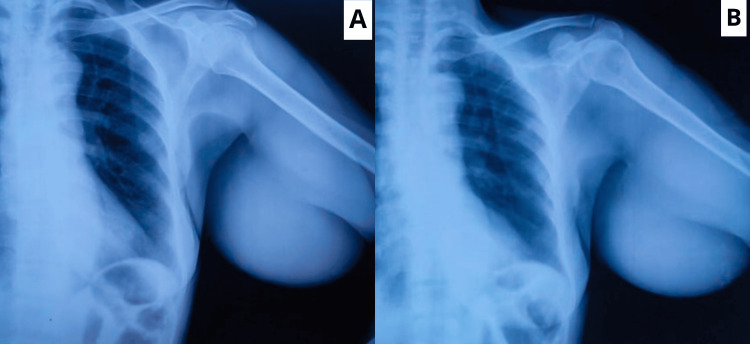
(A) An X-ray of the local region, PA view, showing normal soft tissue and bones. (B) There are no microcalcifications over the swelling. PA: posteroanterior

There was no local rise in temperature on palpation; the swelling was soft in consistency and non-tender without any associated discharge from the scab site, and the swelling had a smooth outline. Axillary lymph nodes were not enlarged. The X-ray of the local region was advised, which showed no obvious abnormality with normal bone and uniformity of the swelling without any microcalcifications, as shown in Figure 2.

Ultrasonography of the affected region also revealed similar findings. The results of fine needle aspiration cytology (FNAC) were in favor of a lipomatous lesion. The patient was scheduled for surgery on an elective basis, and local excision of the swelling was planned under general anesthesia after obtaining consent.

**Figure 3:**
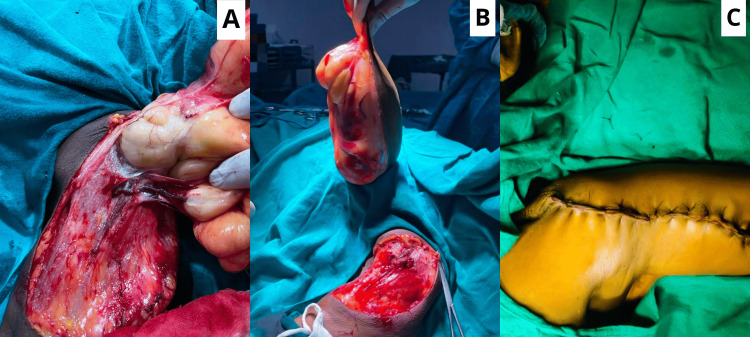
(A) An intraoperative picture showing the surgical excision of the swelling being done and hemostasis being achieved. (B) An intraoperative picture showing the swelling excised as a whole. (C) An intraoperative picture showing the wound being sutured using nylon 2-0 sutures.

Intraoperatively, after the inductions, the swelling was excised from the margins, and a 22×14 cm swelling was noted in the upper arm region of the left upper limb. The swelling was superficial and did not involve or lie in between the muscular planes. There was no infiltration noted in the local tissue as the swelling was excised easily by achieving the surgical plane, hemostasis was achieved, and surgical wound closure was done using nylon 2-0 sutures as shown in Figure 3.

The patient was shifted to postoperative care following surgery. She did well during this period and was discharged on day 2 following the surgery. She was followed up for the examination of the surgical wound, and the follow-up period was uneventful.

**Figure 4:**
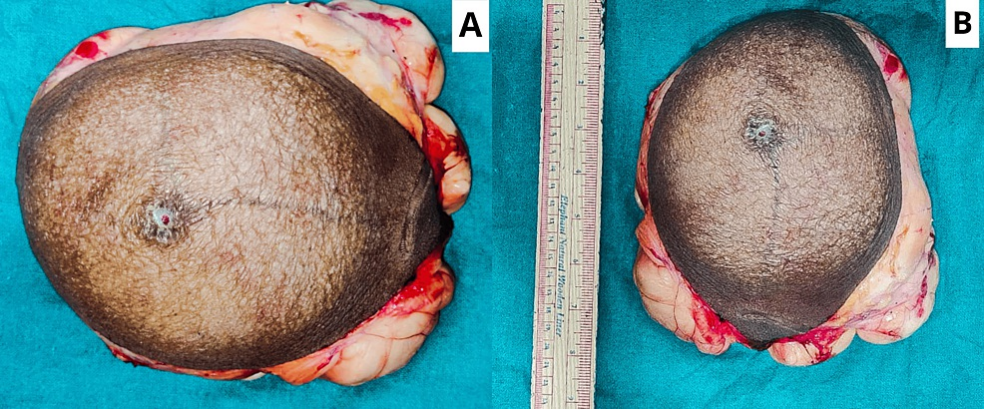
A cut and resected specimen measuring 22×14 cm from the left upper arm following the procedure: (A) horizontal alignment of the resected specimen and (B) vertical alignment of the resected specimen.

The resected specimen was sent for histopathological examination, which revealed a giant lipomatous lesion without any obvious evidence of malignancy as shown in Figure 4.

## Discussion

Ectopic breast tissue develops when the embryonic mammary ridge, a thickening of the ectoderm from the axilla to the groin on either side, fails to resolve. It is the phrase used to refer to both abnormal and supernumerary breast tissue, which are two different things [5]. Nipples, areolae, or both are present in supernumerary breasts, and their glandular tissue compositions may vary. The mammary ridge is the location where they are most frequently found; however, they can also be found on the cheek, neck, shoulder, thigh, or buttock. Although it is typically sporadic, familial and inherited propensity have also been documented [6]. This disorder affects 2%-6% of females and 1%-3% of males, with a third of those individuals having multiple areas of supernumerary tissue growth [7].

The prevalence rates differ significantly by ethnicity and gender, ranging from 0.6% for Caucasians to 5% for Japanese females [8]. Kajava first classified supernumerary breasts in 1915, and his system is still in use today. A full breast, comprising glandular tissue, the nipple, and the areola, belongs to class I. Class II lacks the areola and only includes glandular tissue and the nipple. Class III has no nipple and only glandular tissue and areola. The only tissue in class IV is glandular tissue. The nipple and areola are the only parts of class V (pseudomamma) that lack glandular tissue. There is only the nipple in class VI (polythelia). There is only one areola in class VII (polythelia areolaris). The only component of class VIII (polythelia pilosa) is hair [9].

Although it is there at birth, it does not become active until adolescence, pregnancy, or nursing. It manifests as a soft tissue swelling that may or may not have an adjacent areola or nipple [10]. It has been demonstrated how breast neoplasms can grow in glandular tissue that is situated ectopically [11]. Madej et al. describe a unique instance of a 50-year-old female who developed an invasive accessory breast cancer despite getting frequent mammograms [12].

Although asymptomatic ectopic breasts may be treated conservatively, the majority of ectopic breast care involves surgery [13]. For cosmetic reasons and to prevent any potential consequences, excision is advised in cases of large-sized tissue.

A different method of tumescent liposuction was discovered that enables patients to avoid the central depression appearance that is frequently left behind after the usual ways of resecting the accessory breast tissue [14]. With this alternate treatment, less scarring and a better shape may be achieved than with traditional techniques [15]. Attempts to remove accessory breast tissue might result in surgical problems such as uneven contours, seromas, and perhaps recurrence, regardless of the approach utilized [16].

Differential diagnoses for a mass in the axillary region include the axillary tail of Spence, accessory breast tissue, lymphadenopathy (owing to neoplastic, inflammatory, or viral etiology), lipoma, neuroma, skin lesions (different cystic and tumorous lesions), and skin lesions. In our case, the histology eventually verified what FNAC had first suggested as a giant lipoma, while the clinical impression was in favor of an accessory breast.

Similar to a normal breast mass, the diagnostic and treatment processes for tumors in accessory breast tissue apply in this instance. Due to its rarity and lack of suspicion, the diagnosis could be delayed or even overlooked, which would make receiving prompt treatment more challenging. When tumors or nodules are discovered along the mammary line, the study should take breast tissue into account. FNAC is advised as a quick and economical treatment to eliminate the other alternative diagnosis previously described, to suggest a conclusive diagnosis, and to direct the proper surgical operation.

Supernumerary breasts are a rare condition that can occasionally be difficult to diagnose. Since accessory breast tissue can experience the same pathological changes as the regular breast, including mastitis, fibrocystic disease, and even cancer, an accurate diagnosis is crucial. When evaluating a patient with axillary swelling and accessory breast tissue, other differentials such as lipomas or fibroadenomas must be taken into consideration for early diagnosis and management.

## Conclusions

This example serves as evidence that lipoma may mimic accessory breast tissue, which is just like regular breast tissue in that it can develop neoplasms and other physiological and pathological disease processes. The majority of care is surgical intervention with excision, and FNAC can be performed as a quick and dependable method to aid in early detection. It is advised to get surgery both for cosmetic reasons and to prevent any potential issues in the future.
